# 18 F-Choline-PET/CT for non-FDG-avid salivary gland cancer: a preliminary report

**DOI:** 10.1186/s13550-024-01132-3

**Published:** 2024-07-22

**Authors:** Gregoire B. Morand, Sevda Karimian, Niels J. Rupp, Martin W. Huellner

**Affiliations:** 1 Department of Otolaryngology - Head and Neck Surgery, University Hospital Zurich, Frauenklinikstrasse 24, Zurich, 8091 Switzerland; 2 Department of Pathology and Molecular Pathology, University Hospital Zurich, Zurich, Switzerland; 3 Department of Nuclear Medicine, University Hospital Zurich, Zurich, Switzerland; 4 Faculty of Medicine, University of Zurich, Zurich, Switzerland

**Keywords:** Salivary gland neoplasms, Fluorodeoxyglucose F18, Radiopharmaceuticals, Choline

## Introduction

Malignant salivary gland tumors are rare, accounting for 1–3% of all head and neck cancers [1, 2]. Primary tumors of the salivary gland show a wide variety of morphological entities, with additional subtypes continuously being described [3, 4]. The two most significant prognostic factors are histologic tumor grade and the clinical stage at presentation [5].

Initial staging of malignant salivary gland disease usually involves cross-sectional imaging with contrast-enhanced computed tomography (CT) or magnetic resonance imaging (MRI). Fluoro-desoxy-glucose (FDG) position emission tomography combined with CT or MRI (hybrid FDG-PET) has been increasingly used in salivary gland tumors [6]. However, a subset of primary salivary gland tumors, such as adenoid cystic carcinoma and/or, low-grade salivary gland carcinoma, typically does not exhibit high FDG uptake. Further, FDG uptake alone can be misleading as it can be high in benign entities such as Warthin tumor or benign oncocytic tumors [6].

We therefore felt appropriate to evaluate the potential role of the non-FDG tracer ^18^F-choline in imaging of salivary gland tumors. Our goal was to evaluate the added value of a potentially more specific radiotracer.

## Methods

After obtaining ethics review board approval and informed consent of the patients, we prospectively included patients with clinical and cytological strong suspicion of malignant salivary gland tumors. All patients underwent hybrid FDG-PET followed by hybrid choline-PET. Surgery was offered as a primary treatment modality in resectable cases. Surgical pathology was reviewed by a dedicated head and neck pathologist (NJR) and classified pursuant to the 2017 update of the WHO classification [3].

## Results

The cohort included five prospectively enrolled patients (Table 1). Four cases had cytologically proven malignant (Milan category VI) salivary gland neoplasms. In addition, one case of cytological “salivary gland neoplasm of uncertain malignant potential” (SUMP; Milan category IVb) was included [7].

**Table 1:** Overview of the patients included in the pilot study

	Gender	Age at diagnosis	Tumor site	Cytology Milan category	Histology	Grade	cTNM	pTNM	FDG-PET SUVmax primary tumor	Choline-PET SUVmax primary tumor	Tumor to background ratio (TBR) Choline-PET	FDG-PET SUVmax dominant lymph node	Choline-PET SUVmax dominant lymph node
Pat 1	M	31	Parotid	Milan VI	mucoepidermoid carcinoma	high grade	T2N0M0	T4aN0M0	3.9	8.1	1.43	NA	NA
Pat 2	F	79	Parotid	Milan VI	acinic cell carcinoma	low grade	T2N0M0	T2N0M0	2.2	10.8	1.72	NA	NA
Pat 3	M	91	Parotid	Milan VI	Adenocarcinoma NOS	high grade	T4bN2b M0	NA	2.4	7.2	1.08	2.9	7.4
Pat 4	M	41	Submandibular	Milan VI	adenoid cystic carcinoma	NA	T1N0M0	T2N0M0	3.1	7.8	1.37	NA	NA
Pat 5	M	75	Parotid	Milan IVb	cellular pleomorphic adenoma	NA	NA	NA	2.0	5.5	0.95	NA	NA

cTNM: clinical TNM Staging. pTNM: pathological TNM staging. FDG-PET: flurodesoxyglucose positron emission tomography. SUVmax: maximum standardized uptake value. M: Male, F: Female. NA: not applicable

Tumor-to-background ratio (TBR) was defined as SUVmax of primary tumor divided by SUVmean of contralateral gland

On final pathology, one case was a parotid gland high-grade mucoepidermoid carcinoma, one case of classical (low-grade) acinic cell carcinoma, one case of high-grade adenocarcinoma NOS, which would be classified as (high-grade) mucinous adenocarcinoma according to the forthcoming WHO 2023 classification. The other malignant case was an adenoid cystic carcinoma of the submandibular gland (Table 1).

**Fig. 1:**
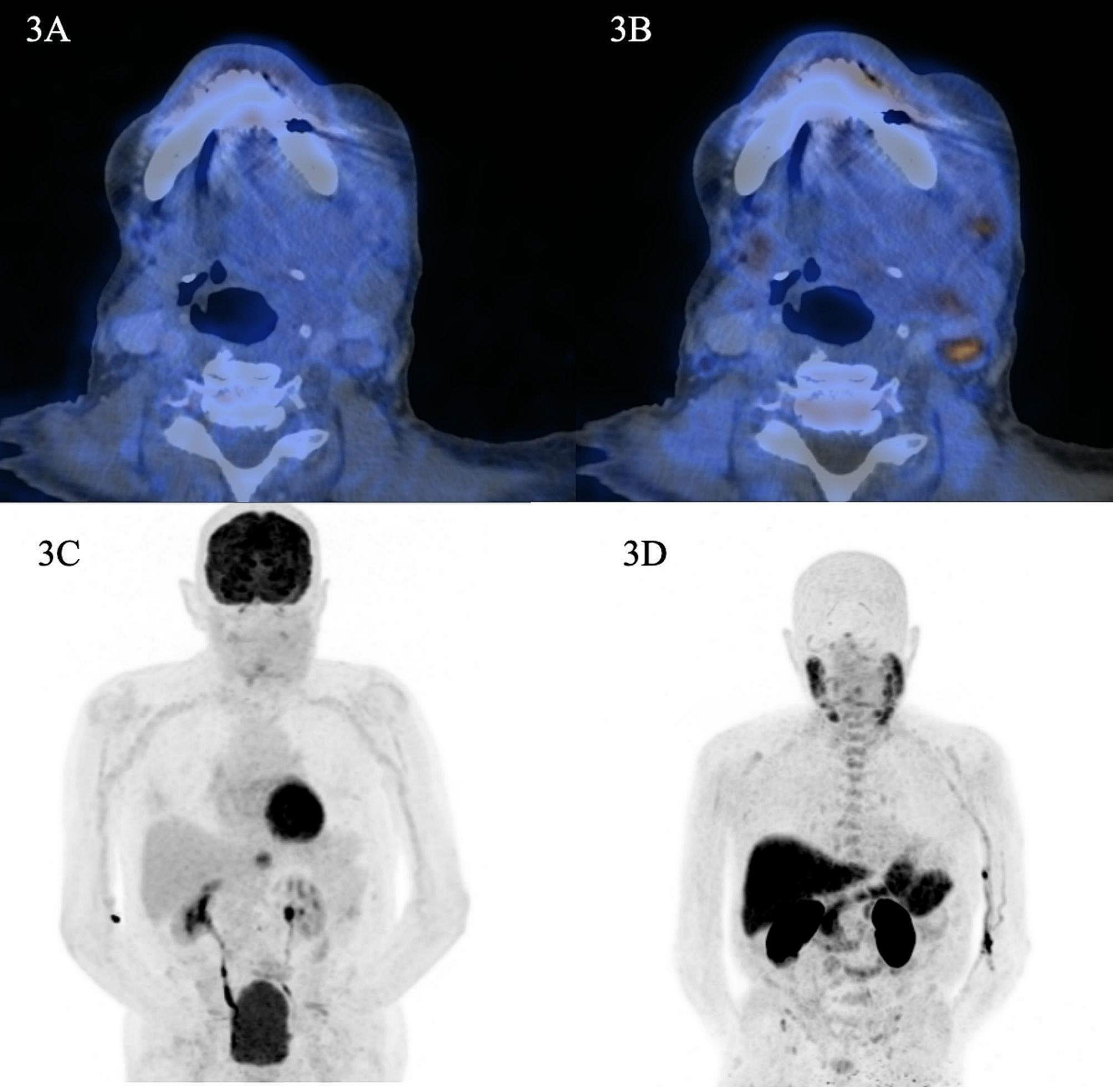
(**A**) axial hybrid FDG-PET/CT images of patient 3 with high grade adenocarcinoma of the left parotid showing low uptake of the tumor (SUVmax 2.4). (**B**) axial hybrid choline-PET/CT images of patient 3 with high grade adenocarcinoma of the left parotid, showing moderate uptake of tumor (SUVmax 7.2). (**C**) maximum intensity projection (MIP) of patient 3 on FDG-PET showing almost no uptake in the parotid gland (and typical uptake in the brain, heart, and lower urinary tract). (**D**) maximum intensity projection (MIP) of patient 3 on choline-PET showing uptake in salivary glands (and in the tumor) and choline uptake in the liver, pancreas, kidney, and spleen

**Fig. 2:**
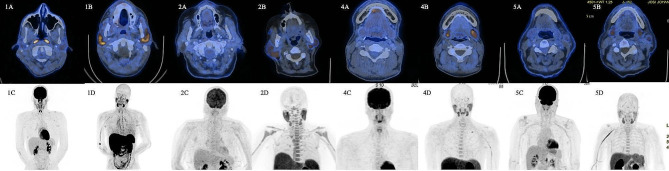
**1A**-**1D**: Axial hybrid FDG-PET/CT (**1A**), axial hybrid choline-PET/CT (**1B**), maximum intensity projection (**1C**) on FDG-PET, and maximum intensity projection of choline-PET (1D) of patient 1 with high-grade mucoepidermoid carcinoma of left parotid **2A**-**2D**: Axial hybrid FDG-PET/CT (**2A**), axial hybrid choline-PET/CT (**2B**), maximum intensity projection (**2C**) on FDG-PET, and maximum intensity projection of choline-PET (**2D**) of patient 2 with low-grade acinic cell carcinoma of right parotid **4A**-**4D**: Axial hybrid FDG-PET/CT (**4A**), axial hybrid choline-PET/CT (**4B**), maximum intensity projection (**4C**) on FDG-PET, and maximum intensity projection of choline-PET (**4C**4D) of patient 4 with adenoid cystic carcinoma of left submandibular gland **5A**-**5D**: Axial hybrid FDG-PET/CT (**5A**), axial hybrid choline-PET/CT (**5B**), maximum intensity projection (**5C**) on FDG-PET, and maximum intensity projection of choline-PET (**5D**) of patient 5 with pleomorphic adenoma of left parotid gland

All malignant cases showed low uptake of the primary tumor on FDG-PET (average SUVmax 2.9), while the uptake was higher on choline-PET (mean SUVmax 8.5) (see Figs. 1 and 2). One case had positive nodal disease and the nodal uptake was higher in hybrid choline-PET than in hybrid FDG-PET (7.4 vs. 2.9, respectively).

Interestingly, the uptake of the primary tumor was stronger in choline PET than FDG-PET. In particular, the case of adenoid cystic carcinoma also showed relative high choline uptake, even though adenoid cystic carcinoma are known to be frequently non-FDG-avid [6]. However, also the background uptake by physiologic salivary gland tissue was considerably higher with choline PET compared to FDG-PET.

The fifth case was a patient with a salivary gland neoplasm of uncertain malignant potential (SUMP) who underwent FDG-PET and choline PET, in which final pathology revealed a cellular pleomorphic adenoma. The choline SUVmax of the tumor was 5.5, which, albeit lower than in malignant tumors, remains comparable to these (mean SUVmax 8.5).

## Discussion

Our limited data represents, to the best our knowledge, the first description of the potential use of hybrid choline-PET in salivary gland neoplasms. We chose choline as it is incorporated in cell membrane phospholipids through phosphorylation [8]. Malignant transformation of cells is associated with induction of choline kinase activity, whereas phosphoryl choline is thought to be present at low or undetectable levels in normal tissues [8]. Although our data shows that choline was somewhat superior to FDG for salivary gland cancer, especially low-grade tumors and adenoid cystic carcinoma, mainly owing to the comparably high physiologic background uptake that impacts the assessment of the primary tumor, the imaging results were not strongly convincing for us to continue our endeavour for these patients, or encourage other groups to test choline-PET for this purpose. Future works may, however, address the issue of nodal staging or test the potential use of choline-PET for detection of distant metastasis of salivary gland cancer. It is important to report our limited experience, as more appropriate radiotracers are needed for salivary gland neoplasms, particularly for adenoid cystic carcinoma, in which FDG-PET typically performs poorly [6].

Other non-FDG radiotracer have been evaluated for salivary gland tumors. Prostate-specific membrane antigen (PSMA), which has broad clinical application in prostate cancer, is known to accumulate in normal salivary gland tissue [9]. Some authors reported stronger uptake in salivary gland tumors than “normal” salivary gland tissue [10]. In addition, the use of PSMA PET and PSMA radioligand therapy (177Lu-PSMA-617) has been reported for detection and treatment of metastatic adenoid cystic carcinoma; however the radioligand therapy effects were limited [11, 12]. In addition, the use of PSMA-targeting radiopharmaceuticals could be further restricted due to their toxicity to the salivary glands. Another novel group of radiotracers that showed encouraging results in salivary gland carcinoma are the fibroblast activation protein inhibitors (FAPI) [13].

## Conclusion

Novel, more specific radiopharmaceuticals are needed in patients with some types of primary salivary gland carcinoma. Our report shows limited benefit of the use of choline over fluoro-desoxy-glucose for hybrid PET imaging in salivary gland malignancies.

## Data Availability

The datasets generated for this study can be obtained upon reasonable request by email to the corresponding author.

## Abbreviations

CT: Computed tomography
FAPI: Fibroblast activation protein inhibitors
FDG: Fluoro-desoxy-glucose
MRI: Magnetic resonance imaging
PET: Position emission tomography
PSMA: Prostate-specific membrane antigen
SUMP: Salivary neoplasm of unknown malignant potential
WHO: World health organization
